# Testicular sperm extraction for men with cryptozoospermia – are we jumping the gun?

**DOI:** 10.1080/20905998.2024.2367333

**Published:** 2024-06-12

**Authors:** Andrian Japari, Baris Altay, Wyns Christine, Selahittin Cayan, Ramadan Saleh, Rupin Shah, Ashok Agarwal

**Affiliations:** a Fertility Clinic, Telogorejo Hospital, Semarang, Indonesia; b Global Andrology Forum, Moreland Hills, OH, USA; c Urology Department, Ege University, Izmir, Turkey; d Cliniques Universitaires Saint-Luc, Brussels, Belgium; e Urology Deparment, Mersin University, Mersin, Turkey; f Department of Dermatology, Venereology and Andrology, Faculty of Medicine, Sohag University, Sohag, Egypt; g Ajyal IVF Center, Ajyal Hospital, Sohag, Egypt; h Division of Andrology, Department of Urology, Lilavati Hospital and Research Centre, Mumbai, India; i Cleveland Clinic Foundation, Cleveland, OH, USA

**Keywords:** Cryptozoospermia, ICSI, Male infertility, microTESE, Sperm, Testis

## Abstract

Cryptozoospermia is a condition where spermatozoa can only be detected with extensive sperm-pellet analysis after routine semen analysis failed to detect any spermatozoa. Intracytoplasmic sperm injection (ICSI) can help these cryptozoospermic patients overcome their infertility issues. ICSI can be done with ejaculated or testicular sperm obtained by surgical sperm retrieval (SSR) techniques, each with its advantages and disadvantages. In managing cryptozoospermic patients, there is a need to first investigate the possibility of improving sperm numbers and quality based on etiologies. SSR is a part of the management, but presents also potential risks. The benefit of SSR is that sperm obtained by SSR in cryptozoospermic patients tend to give better outcomes when used for ICSI. However, besides the physical burden, SSR also has potential drawbacks, such as psychological trauma associated with surgery in some patients, the need for extra funding for the surgery, and immaturity of the sperm. According to recent studies, ICSI using testicular sperm from cryptorchid patients provides better pregnancy and live birth rates, as well as a lower miscarriage rate. Some studies contradict these findings, hindering the possibility of providing a robust answer on this matter. This review aims to present ICSI outcomes with ejaculated or testicular sperm, taken from cryptozoospermic patients, and discuss the results of comparative studies highlighting the possible reasons behind these discrepancies, and eventually offer an expert opinion for clinicians.

## Introduction

Cryptozoospermia is a condition where spermatozoa cannot be found in the ejaculate after routine semen analysis, but are detected after extensive examination of the sperm pellet following centrifugation [1]. This condition is also called virtual azoospermia [2]. It leads to severe male factor infertility, although with a better prognosis than azoospermia. The prevalence of cryptozoospermia was approximately 8.73% according to the results of three infertility clinics in Turkey from 2011 to 2016 among all their infertile men [3]. However, this figure may vary according to patient demographics and comorbidities.

It may be difficult to distinguish cryptozoospermia from absolute azoospermia. Indeed, as the detection of spermatozoa in the ejaculate is affected by many factors such as temperature, skills of andrology lab technicians, centrifugation and sampling, it is recommended to analyze at least two pellets on two separate/different occasions [1,4–6]. The presence of a single spermatozoon confirms the diagnosis of cryptozoospermia thus excluding azoospermia [1]. Many etiological factors of cryptozoospermia have been reported, such as high-grade varicocele, hormonal abnormalities, genetic abnormalities, infections, radiation, chemotherapy, cryptorchidism, and testicular tumors. Cryptozoospermia is usually caused by a spermatogenic disorder, while its association with obstructive etiologies is less common [7]. Currently, no effective treatment for improving spermatogenesis in cryptozoospermic patients has been established, and often different drugs such as selective estrogen receptor modulators, aromatase inhibitors, or antioxidants are prescribed [7], while the evidence to support these therapies is either very limited or absent [8].

Today, intracytoplasmic sperm injection (ICSI) is considered the first-choice therapy for cryptozoospermia [9], and often these infertile cryptozoospermic men are recommended to undergo surgical sperm retrieval (SSR). However, with a skilled evaluation of the sperm sample, motile ejaculated sperm might be used instead of testicular sperm for ICSI [9].

Debates around the use of testicular sperm raise the question of whether ejaculated sperm might be exposed to higher oxidative stress compared to testicular sperm, and if they may negatively influence sperm quality and ICSI success rates [2,10].

Additionally, surgical risks and outcomes need to be considered. With regards to SSR for cryptozoospermia, there are several techniques available, such as testicular fine needle aspiration (TfNA), testicular sperm extraction (TESE), testicular sperm aspiration (TESA)/testicular core biopsy (TCE), and microdissection testicular sperm extraction (microTESE) to obtain testicular sperm for ICSI [11,12]. TESE was first reported by Devroey et al. [13] for azoospermic patients in 1995. Then, Schlegel [14] described a new technique, based on the idea that most men with NOA have heterogeneity within their seminiferous tubules and may demonstrate isolated foci of spermatogenesis that can be retrieved microsurgically, using a microscope with a 25-fold optical magnification. Despite its high cost for equipment, longer operative time, and need for surgeons skilled in microsurgical techniques, high sperm retrieval rates have been reported [12,14–16]. The overall success rate of retrieving sperm with these techniques varies between 25% and 50% in most reports. The probability of finding sperm is different for each technique, with microTESE having the highest probability reported to be 10% higher than with other techniques, followed by TESE, and TESA having the lowest probability [17–21].

Aside from benefits, SSRs also come with the risks of damaging testicular integrity. The impact of testicular damage depends on the type of SSR procedure. TESE had the greatest impact, followed by TESA, and lastly, the least damage is with microTESE [19,20] which minimizes testicular damage by detecting and orienting the sampling towards the most dilated and opaque tubules [12,15]. The testicular damage from SSR techniques may cause testicular fibrosis which may convert the patients from cryptozoospermia to azoospermia.

The aim of this review is to examine the ICSI outcomes with ejaculated and testicular sperm in cryptozoospermic patients and discuss the possible reasons behind the differences.

## Methods

This review was constructed from original studies, systematic reviews, and meta-analyses that were published in the last 10 year from 1 January 2013 until 31 December 2023. The search for articles was done on 10 January 2024 using PubMed and Scopus electronic databases.

For PubMed, the search terms were: ((‘cryptozoosperm*’[All Fields] OR ‘cryptosperm*’[All Fields]) AND (‘ejaculated sperm’[All Fields] OR ‘testicular sperm’[All Fields] OR ‘surgical’[All Fields] OR ‘ICSI’[All Fields])) AND (2013:2023[pdat]). For Scopus searches, we used the search terms: ALL (‘cryptozoosperm*’ OR ‘cryptosperm*’) AND ALL (‘ejaculated sperm’ OR ‘testicular sperm’ OR ‘surgical’ OR ‘ICSI’) AND PUBYEAR > 2012 AND PUBYEAR < 2024. Moreover, the authors (AJ, BA, WC) also conducted citation searching of studies to select any relevant articles.

The inclusion criteria were: (1) original studies; (2) any design; (3) published from 1 January 2010 through 31 December 2023; (4) assessed ‘ICSI’ and ‘ejaculated sperm/testicular sperm’; and (5) included patients of any age and ethnicity. Only studies containing original research on the outcome of ICSI with testicular sperm and/or ejaculated sperm in cryptozoospermic patiens. Exclusion criteria included non-English articles, review articles, editorials/correspondence, or commentaries, and studies that did not include cryptozoospermia and the use of ejaculated sperm and/or testicular sperm.

The assessment of quality of the included studies in the current review (Table 1) showed that most studies had a low risk of bias except for two studies that had high risk.

**Table 1.** Assessment of quality of included studies*.

Study	Selection	Comparability	Outcome	Total	Risk of Bias
Ben-ami, et al. 2013 [22]	***	*	***	7	Low
Cui, et al. 2017 [23]	***	*	***	7	Low
Saito, et al. 2018 [10]	* * *	*	* * *	7	Low
Hernandez-Nieto, et al. 2019 [24]	* * *	*	* * *	7	Low
Hernandez-Nieto, et al. 2019 [25]	* * *	*	* * *	7	Low
Alharbi, et al. 2021 [26]	* * *	—	* * *	6	High
Hibi, et al. 2023 [27]	* * *	*	* * *	7	Low
Kaiyal, et al. 2023 [28]	* * *	—	* * *	6	High
Marinaro, et al. 2023 [9]	* * *	*	* * *	7	Low

*The Newcastle Ottawa Scale [29]; The meta-analyses by Abhyankar, et al.. (2016) [30] and Esteves, et al.. (2017) [31] are not assessed.

## Results

### Assisted reproductive technology (ART) outcomes in cryptozoospermic men using ejaculated or testicular sperm

ICSI is commonly performed with ejaculated sperm, because of the easiness in obtaining the samples [32] and as ejaculated spermatozoa have usually finished the maturation process, yielding high success rates in ICSI treatment cycles [33]. Before deciding to use ejaculated sperm for ICSI, sperm can also be assessed for various parameters such as sperm concentration, motility, morphology [1], sperm deoxyribonucleic acid (DNA) fragmentation (SDF) [34]. Additional procedures to ICSI with ejaculated sperm may also be applied with a view to improving the selection of the sperm cells, such as the use of microfluidic sperm sorting [35], especially in the presence of high SDF [36,37].

The SSR procedure, which is usually carried out when a patient is azoospermic, may also be considered when ejaculated sperm is available after multiple previous ICSI failures, if prior measures to reduce SDF have been proposed [38]. The level of sperm maturation in the testicles varies greatly, from immature to mature with higher proportions of immature sperm with potential impact on ICSI outcomes [39,40]. The benefit of using testicular sperm was most often reported when SDF was high [17,23,31].

In men with cryptozoospermia, ICSI may be challenged by both the quality and quantity of ejaculated sperm. It is difficult to find spermatozoa in the ejaculates of cryptozoospermic patients and it commonly requires a centrifugation process [9,23,24,30]. However, excessive centrifugation at high speed can induce SDF [30,41].

Studies have demonstrated that cryptozoospermia could negatively affect fertilization and clinical pregnancy rates after ICSI [27,28,42]. This was mainly attributed to the high probability of SDF and chromosomal abnormalities in men with low sperm counts [26].

Besides the possibility of reduced ICSI outcomes, inconsistent presence of sperm in the ejaculate with the risk of finding no sperm on the day of the egg retrieval or having an insufficient number for ICSI may also jeopardize the use of ejaculated sperm. In this case, the SSR has a justification to be carried out as an effort to obtain spermatozoa suitable for ICSI. However, the SSR success rate is not 100%. In cryptozoospermic patients, the SSR success rate by conventional techniques (TESE, TESA) is only 25% − 43% [11,17], while for microTESE, the success rate is 85% − 96% [11,17,43]. Hence, prior to SSR for cryptozoospermic cases, asking for two semen samples on the morning of ovum pick-up (OPU) allows the use of the best specimen either from an ejaculated sample or SSR for ICSI. This information should be conveyed to the patients during consultation before the SSR.

The meta-analysis by Abhyankar et al. [30], including 5 cohort studies, found that ICSI resulted in a similar fertilization rate and pregnancy rate for ejaculated and testicular sperm in patients with cryptozoospermia. They concluded that the available literature does not support a recommendation for cryptozoospermic patients to use testicular sperm over ejaculated sperm.

In contrast, Cui, et al. [23], found differences in the good quality embryo rate, implantation rate, pregnancy rate, and live birth rate between testicular sperm and ejaculated sperm (46.1% vs 36.8%, 52.1% vs 30.7%, 53.6% vs 33.3%, and 44.6% vs 27.1%, respectively), although no difference was observed for fertilization rate (59.6% vs 60.6%). They also stated that testicular sperm are less exposed to contaminants than ejaculated sperm in the urogenital tract, and thus have lower SDF. Their results are in line with those of Saito et al. [10] who found that ICSI with testicular sperm is superior to ICSI with ejaculated sperm in terms of blastocyst formation (47.0% vs 30.8%) and PR (30.7% vs 13.9%), while there was no difference in fertilization rate. The meta-analysis by Kang et al. [2], further confirmed these observations showing higher performance with fresh testicular sperm with regards to good quality embryo rate (relative risk, RR = 1.17; confidence interval, CI 1.05–1.30), implantation rate (RR = 1.52; CI 1.02–2.26), and PR (RR = 1,74; CI 1.20–2.52) and no statistical difference for fertilization rate.

A more recent study found no differences between ICSI with ejaculated and testicular sperm in cryptozoospermic patients for fertilization rate, blastocysts formation, and pregnancy rate, although there were differences in the good quality embryo and canceled cycle rates in favor of testicular sperm [25]. Another study also showed no difference in embryo euploidy rate between both groups [24].

### Controversies on the care of cryptozoospermic patients

Controversial data limit the possibility of providing the clinician with robust recommendations regarding the use of ejaculated or testicular sperm for ICSI in patients with cryptozoospermia. Looking at the main clinically relevant outcomes, some studies are in favor of testicular sperm for pregnancy rates [2,10,22,23] and live birth rates [22,23], while others found similar outcomes between ejaculated sperm and testicular sperm for pregnancy rates [25,30] and live birth rates [27]. Reasons behind discrepancies could be linked to the etiology of cryptozoospermia which is not mentioned in most comparisons as well as numerous other confounders such as female age, issues related to the ovarian stimulation protocol, and availability of sufficient numbers of sperm in ejaculates for performing ICSI on all collected oocytes. Only a few studies did consider the female factors including age, ovarian reserve, and the ovarian stimulation protocol [22,23,27].

A summary of the advantages and limitations, as well as the controversies regarding the use of ejaculated versus testicular sperm in ART for cryptozoospermic patients, is presented in Table 2.

**Table 2.** Summary of the advantages, limitations, and controversies regarding the use of ejaculated versus testicular sperm in ART for cryptozoospermic patients.

Conditions Analyzed	Brief Summary
Sample collection and processing	In favor of ejaculated sperm for easiness to obtain and to process, without additional cost for surgery and the possibility of testicular tissue damage following SSR [21,32]
SDF	Testicular sperm have lower SDF compared to ejaculated sperm [17,23,31]
Fertilization rate	There is no significant difference in fertilization rate between ejaculated sperm and testicular sperm [2,10,23,25,30]
Blastocyst rate	Controversial. Some studies are in favor of testicular sperm [10] while others showed no significant differences between ejaculated sperm and testicular sperm [25]
Good quality embryos	ART using testicular sperm produces more good quality embryos compared to ART using ejaculated sperm [2,23,25]
Embryo euploidy rate	There is no significant difference in embryo euploidy rate between ejaculated sperm and testicular sperm [24]
Implantation rate	ART using testicular sperm has a higher implantation rate compared to ART using ejaculated sperm [2]
Pregnancy rate	Controversial. Some studies showed that ART using testicular sperm had a higher pregnancy rate compared to ART using ejaculated sperm [2,10,22,23], while others showed similar results [25,30]
Live birth rate	Controversial. Some studies showed that ART using testicular sperm had a higher live birth rate compared to ART using ejaculated sperm [22,23], while others showed similar results [27]
Canceled cycles	ART using testicular sperm had a lower canceled cycle compared to ART using ejaculated sperm [25]

ART: Assisted reproductive technology; SDF: Sperm DNA fragmentation; SSR: Surgical sperm retrieval

A well-designed study with one arm using testicular sperm obtained by SSR, and the other arm using ejaculated sperm (provided the numbers are sufficient to perform ICSI on all eggs), taking into account a homogenous female population and men with the etiology of cryptozoospermia could help resolve this debate.

### Case scenario

A cryptozoospermic patient, aged 39 years, and his spouse aged 38 years with normal gyno-endocrinologic evaluation, wish to enroll in the ART program. His body mass index is 29, and had no varicocele on physical examination. His past medical history revealed that there was no record of cryptorchidism, scrotal hernia, testicular trauma, or orchitis. His previous semen analyses prior to entering the ART program showed mostly pellet-negative azoospermia, but cryptozoospermia in some. No chromosome abnormality or gene abnormality was detected. His serum hormonal assessment showed a total testosterone level of 560 mg/dL, follicle-stimulating hormone (FSH) level of 10 mIU/mL, and an estradiol level of 32 pg/mL, with a testosterone/estradiol ratio of 17.5. His serum prolactin level was within normal range (8 ng/mL).

In this case, after the decision to undergo ART with ICSI was established, the couple was encouraged to do SSR to obtain the spermatozoa. This decision was based on the slightly higher maternal age and the inconsistency of finding sperm in most previous semen analyses. The proposed SSR technique was microTESE, hoping to provide a better chance to find sperm with minimal testicular damage, although this technique requires longer operative time and significantly more expense.

Options to repeat ejaculates on the day of the OPU or to vitrify oocytes with successive warming and ICSI on future ejaculates were discussed with the couple because of the possibility of fear of being operated on, or cost issues.

## Conclusions

In cryptozoospermic patients, ICSI can be done utilizing both ejaculated and testicular sperm. Both have their advantages and disadvantages. The advantage of ejaculated sperm is its maturity, while the disadvantage is the difficult process of obtaining it, due to the very low number of sperm present in the ejaculate and the need for centrifugation process which leads to the increase of sperm DNA damage. As for testicular sperm, its advantage is the low level of DNA damage and its disadvantages are the need for SSR which can lead to testicular tissue damage, the cost needed to perform SSR, and the low maturity of the sperm. Every option should be discussed with the patients.

### Key Points

- In cryptozoospermic conditions, SSR can be performed, especially when the presence of ejaculated sperm on the day of ICSI is uncertain and prior cryopreservation has been considered but is not an option in the current cycle. SSR can also be performed if an insufficient number of sperm is available in the ejaculate for one ICSI cycle.
- More robust evidence is needed to use testicular sperm in patients with cryptozoospermia when enough spermatozoa are available in the ejaculate as there are controversial studies regarding the pregnancy and live birth rates, and the quality of the studies is poor.

## Expert opinions

In managing cryptozoospermic patients, the first approach is improving sperm quality based on various etiologies of poor sperm quality e.g. lifestyle modification, weight reduction, varicocele repair, and restoration of hormonal imbalance. A minimum of two separate/different semen samples with sperm pellet analyses should be taken into consideration before deciding on SSR.

SSR may be considered in cryptozoospermia as it showed higher or similar pregnancy and live birth rates, compared to ejaculated sperm especially: 1) if the presence of ejaculated sperm on the day of the ICSI cycle is uncertain and prior cryopreservation has been considered but is not an option; 2) if an insufficient number of sperm is available in the ejaculate for one ICSI cycle; 3) in case of previous ICSI failure with ejaculated sperm. However, besides the possibility of surgical complications, this procedure also has other potential drawbacks, such as psychological trauma from surgery in some patients, the need for extra funding for the surgery, and the lower maturity of the sperm.

Other options to be discussed with the patient are the possibility of undergoing ICSI with repeated ejaculates on the day of the OPU, using previously cryopreserved samples, or opting for oocyte vitrification with successive warming and ICSI with future fresh ejaculates.

## Abbreviations list

ART: Assisted reproductive technology
CI: Confidence interval
DNA: Deoxyribonucleic acid
FSH: Follicle stimulating hormone
ICSI: Intracytoplasmic sperm injection
microTESE: Microdissection testicular sperm extraction
NOA: Non-obstructive azoospermia
OPU: Ovum pick-up
RR: Relative risk
SDF: Sperm DNA fragmentation
SSR: Surgical sperm retrieval
TCE: Testicular core biopsy
TESA: Testicular sperm aspiration
TESE: Testicular sperm extraction
TfNA: Testicular fine needle aspiration
